# Superconductivity emerging from a suppressed large magnetoresistant state in tungsten ditelluride

**DOI:** 10.1038/ncomms8804

**Published:** 2015-07-23

**Authors:** Defen Kang, Yazhou Zhou, Wei Yi, Chongli Yang, Jing Guo, Youguo Shi, Shan Zhang, Zhe Wang, Chao Zhang, Sheng Jiang, Aiguo Li, Ke Yang, Qi Wu, Guangming Zhang, Liling Sun, Zhongxian Zhao

**Affiliations:** 1 Institute of Physics and Beijing National Laboratory for Condensed Matter Physics, Chinese Academy of Sciences, Beijing 100190, China; 2 Shanghai Synchrotron Radiation Facilities, Shanghai Institute of Applied Physics, Chinese Academy of Sciences, Shanghai 201204, China; 3 State Key Laboratory for Low dimensional Quantum Physics, Department of Physics, Tsinghua University, Beijing 100084, China; 4 Collaborative Innovation Center of Quantum Matter, Beijing 100190, China

## Abstract

The recent discovery of large magnetoresistance in tungsten ditelluride provides a unique playground to find new phenomena and significant perspective for potential applications. The large magnetoresistance effect originates from a perfect balance of hole and electron carriers, which is sensitive to external pressure. Here we report the suppression of the large magnetoresistance and emergence of superconductivity in pressurized tungsten ditelluride via high-pressure synchrotron X-ray diffraction, electrical resistance, magnetoresistance and alternating current magnetic susceptibility measurements. Upon increasing pressure, the positive large magnetoresistance effect is gradually suppressed and turned off at a critical pressure of 10.5 GPa, where superconductivity accordingly emerges. No structural phase transition is observed under the pressure investigated. *In situ* high-pressure Hall coefficient measurements at low temperatures demonstrate that elevating pressure decreases the population of hole carriers but increases that of the electron ones. Significantly, at the critical pressure, a sign change of the Hall coefficient is observed.

## Abstract (web summary)

Tungsten ditelluride has been recently discovered to possess very large and unsaturated magnetoresistance, which opens interesting perspectives for potential applications. Here the authors show suppression of the magnetoresistance and emergence of superconductivity at high pressure.

## Graphical abstract

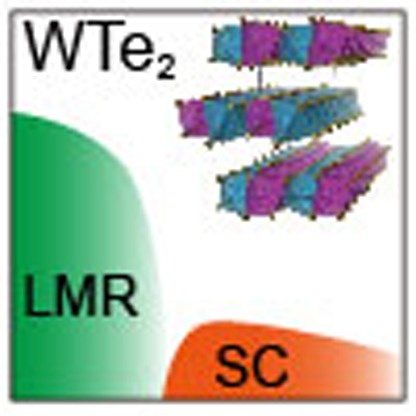

Tungsten ditelluride (WTe_2_) is a well-known non-magnetically thermoelectric semimetal^1^,^2^,^3^. Recently, its unexpected property of large magnetoresistance (LMR) has been discovered^4^, yielding a research hotspot in condensed matter physics and material science. The LMR effect found in WTe_2_ and some other nonmagnetic compounds^4^,^5^,^6^,^7^,^8^,^9^,^10^ is a peculiar transport property. At ambient pressure, WTe_2_ crystallizes in a crystal structure of distorted MoS_2_ (ref. 11), and the distortion is induced by the tungsten chains that are arranged along the *a* axis of the orthorhombic unit cell. The electrical resistance along the *a* axis increases markedly when magnetic field is applied perpendicularly to the dichalcogenide layers (along the *c* axis), resulting in the LMR effect. Theoretical and experimental investigations indicate that the LMR effect is resulted from the perfect balance between electron and hole Fermi pockets along the Γ–X direction in the Brillouin zone^4^,^12^,^13^, different from that of giant and colossal magnetoresistance previously found in magnetic materials^14^,^15^. Due to the complicated band structure with multiple Fermi pockets in WTe_2_, the fine details of the electronic structure play a significant role in the LMR effect. It is well known that pressure as an important control parameter can effectively tune lattice structures and the corresponding electronic states in a more systematic fashion, avoiding the complexity brought by chemical doping^13^,^16^,^17^,^18^,^19^,^20^. In particular, the Fermi surface changes had been observed in pressurized NbSe_2_ and NbS_2_ (ref. 21 and ref. 22, respectively). Therefore, the high-pressure studies on WTe_2_ are important to explore novel phenomena and understand the physics behind.

We demonstrate here that how external pressure suppresses the LMR state and induces the emergence of superconductivity in WTe_2_ with the experimental results from multiple *in situ* high-pressure measurements. We find that, upon increasing pressure, the inter-plane lattice constant *c* is substantially reduced without structural phase transition, while the LMR effect is gradually suppressed and turned off at a critical pressure of 10.5 GPa where superconductivity accordingly emerges. The superconducting transition temperature (*T*_c_) reaches 6.5 K at ∼13.0 GPa and then decreases monotonically with increasing pressure down to 2.6 K at ∼24.0 GPa. Significantly, our Hall measurements reveal a sign change in the Hall coefficient at the critical pressure, indicating a quantum phase transition of the Fermi surface reconstruction.

During the preparation of this manuscript, we became aware of an arXiv paper that reports the evidence of pressure-induced superconductivity in WTe_2_ from electrical resistance measurements without a pressure-transmitting medium^23^.

## Results

### Structure under pressure

**Figure 1:**
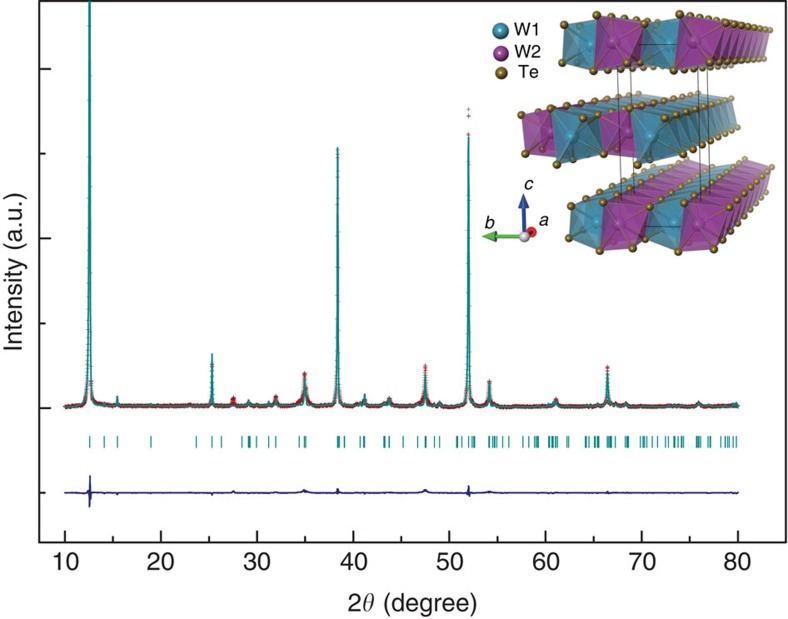
Experimental and indexed X-ray diffraction patterns of WTe_2_ at ambient pressure. The purple crosses represent experimental data, and the cyan line and bars represent calculated Bragg reflection pattern and positions. The inset shows the three-dimensional layer structure constructed by an edge-shared WTe_6_ octahedron.

**Figure 2:**
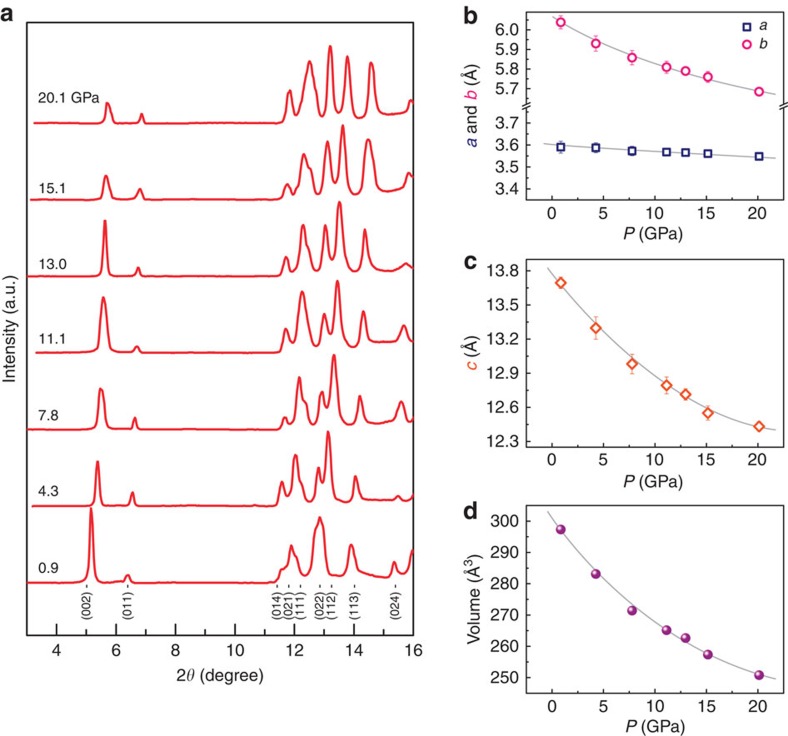
Structure information of WTe_2_ sample at high pressure. (**a**) X-ray diffraction patterns of WTe_2_ collected at different pressures, showing no crystal structure transition. (**b**,**c**) Pressure dependence of lattice constants (*a*, *b* and *c*). The error bars represent s.d. (**d**) Volume change as a function of pressure.

We first characterize the structure of the WTe_2_ sample at ambient pressure. Figure 1a shows the X-ray diffraction (XRD) pattern of a powdered sample that is ground from a few pieces of single crystals. As it can be seen, the Bragg peaks in the pattern can be well indexed by orthorhombic structure. To clarify whether there is a structure change in pressurized WTe_2_, we perform *in situ* high-pressure synchrotron XRD measurements. The results shown in Fig. 2a indicate no first-order structure phase transition under pressure up to 20.1 GPa. Then we extract the lattice parameters as a function of pressure, shown in Fig. 2b,c. The pressure dependence of volume also displays in Fig. 2d. It is found that the lattice constants (*a*, *b* and *c*) as well as the volume decrease continuously upon increasing pressure. However, the reduction of *c* is substantial, compared with those of the in-plane parameters.

### Pressure-induced superconductivity

**Figure 3:**
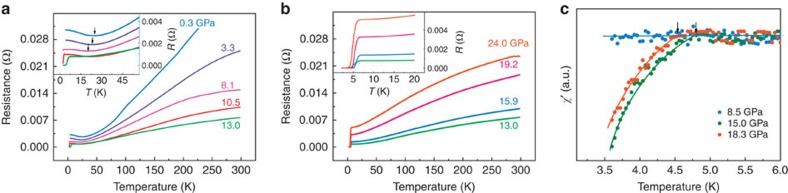
Electrical resistance and susceptibility features of single-crystal WTe_2_ at different pressures. (**a**) The plot of electrical resistance as a function of temperature measured without magnetic field for the pressures ranging from 0.3 to 13.0 GPa. The inset displays the enlarged view of the low-temperature electrical resistance, where a characteristic temperature is defined by the minimum resistance and is taken as turn-on temperature (T*_ZF_) of the LMR effect, as indicated by arrows. (**b**) Temperature dependence of electrical resistance measured at different pressures between 13.0 and 24.0 GPa. The inset clearly shows electrical resistance drops and zero-resistance behaviour. (**c**) The real part of the a.c. magnetic susceptibility (*χ′*) versus temperature for the single-crystal WTe_2_ at different pressures, which confirms the diamagnetism at the selected pressures of 15.0 and 18.3 GPa, respectively. The arrows indicate the onset temperatures of the superconducting transitions.

The electrical resistance measurement for the single crystal is performed under quasi-hydrostatic pressure. Since WTe_2_ has an anisotropic electron structure, the LMR effect was discovered along the tungsten chain direction (*a* axis)^4^. To reveal the pressure effect on the LMR state, we apply the magnetic field and current in the same manner as ambient pressure. Figure 3 shows the typical temperature dependence of electrical resistance measured at zero magnetic field for pressures ranging from 0.3 to 24.0 GPa. In Fig. 3a, the electrical resistance curve at 0.3 GPa shares the similar behaviour to that measured at ambient pressure^4^,^13^. Upon increasing pressure below 13.0 GPa, the electrical resistance in the whole temperature range is suppressed, while it is enhanced above 13.0 GPa. Intriguingly, at the pressure of 10.5 GPa, the resistance drops abruptly at 2.8 K. Such a drop becomes more pronounced at higher pressures, and the zero-electrical resistance is achieved between 11.0 and 24.0 GPa (Fig. 3b).

To confirm whether the zero-electrical resistance state is superconducting or not, we carry out *in situ* high-pressure alternating-current (a.c.) susceptibility measurements. The Meissner effect is observed at the selected pressures of 15.0 and 18.3 GPa, respectively (Fig. 3c). The onset temperatures of the diamagnetism are consistent with that of the electrical resistance drop (Fig. 3b). Both electrical resistance and magnetic measurements coordinately confirm that pressure induces a superconducting transition in WTe_2_. The *T*_c_ can reach to 6.5 K at ∼13.0 GPa and monotonically decreases down to 2.6 K at ∼24.0 GPa.

### Suppression of the LMR state

**Figure 4:**
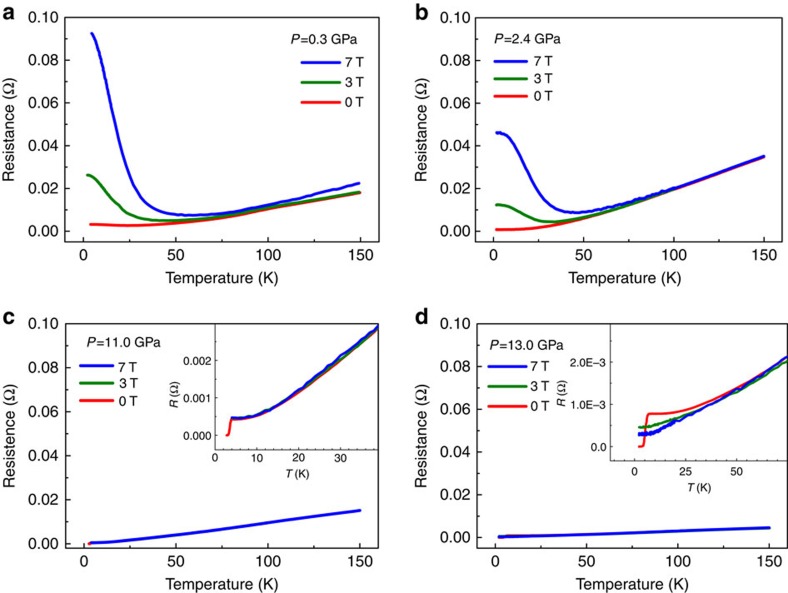
High-pressure electrical resistance versus temperature under different magnetic fields. (**a**,**b**) The electrical resistance as a function of temperature at 0.3 and 2.4 GPa, respectively, illustrating the obvious suppression of the LMR effect by increasing pressure. (**c**) The plot of resistance versus temperature at 11.0 GPa. The inset shows an enlarged view of a full suppression of the positive magnetoresistance and a zero resistance at 3.2 K. (**d**) The temperature dependence of electrical resistance at 13.0 GPa. The inset displays the elimination of superconductivity by magnetic fields.

To reveal how the LMR state evolves into the superconducting state, we systematically investigate the temperature dependence of electrical resistance at fixed pressures under different magnetic fields. We find that the positive LMR effect of WTe_2_ is suppressed by applying pressure (Fig. 4a,b). At the pressure of 11.0 GPa and above, the positive magnetoresistance effect no longer exists (Fig. 4c,d), and the superconductivity appears simultaneously.

### Determination of upper critical magnetic field

**Figure 5:**
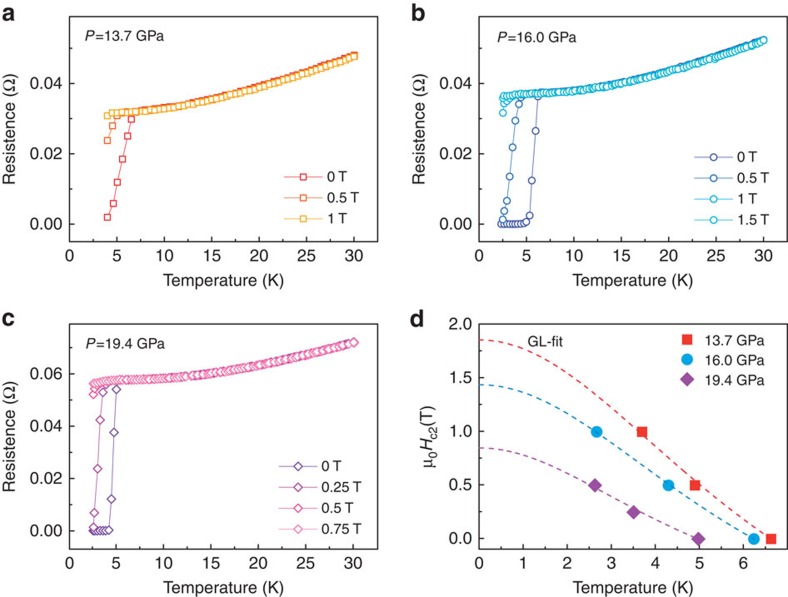
Determination of the value of upper critical field (*H*_c2_) for the superconducting WTe_2_. (**a**–**c**) Temperature dependence of electrical resistance at fixed pressures under different magnetic fields. (**d**) *H*_c2_ as a function of temperature. The dashed lines represent the Ginzburg–Landau (GL) fits.

Notably, the superconductivity of the sample is fully suppressed at 13.0 GPa under 3 and 7 Tesla (Fig. 4d). To determine the value of upper critical magnetic field (*H*_c2_) precisely, we perform the temperature dependence of resistance measurements under lower magnetic fields on the pressurized sample (Fig. 5a–c). Using the Ginzburg–Landau formula to fit the experimental data yields the values of upper critical magnetic field at zero temperature: 1.86 T at 13.7 GPa, 1.44 T at 16.0 GPa and 0.85 T at 19.4 GPa (Fig. 5d).

### Pressure–temperature phase diagram

**Figure 6:**
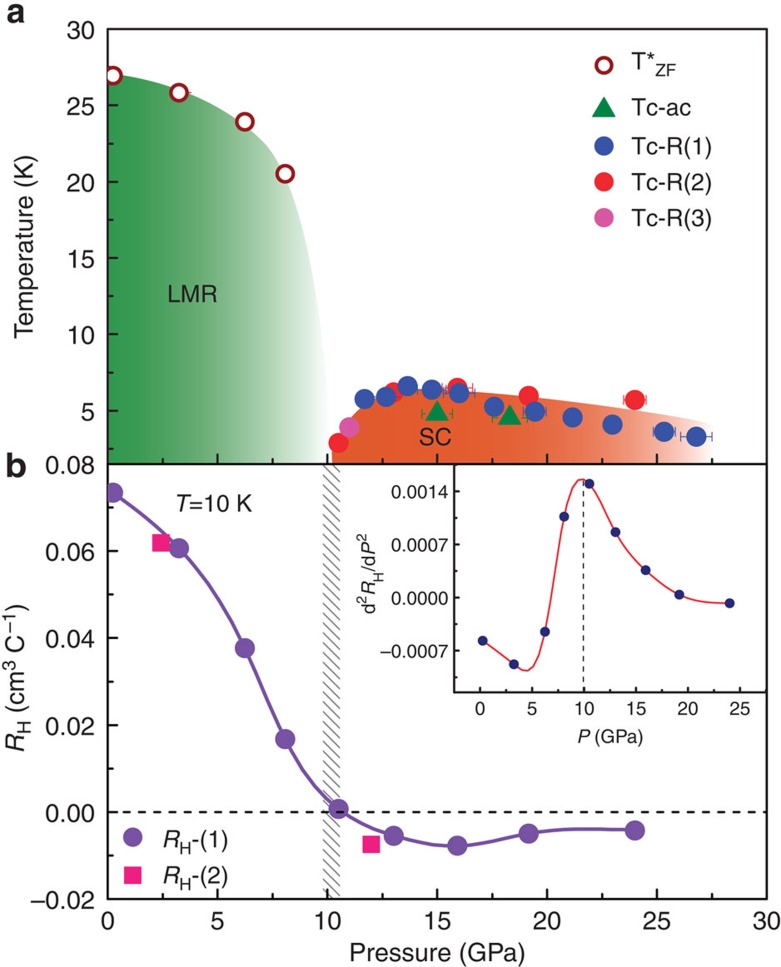
Pressure–temperature phase diagram of WTe_2_ and pressure-dependent Hall coefficient. (**a**) The T*_ZF_ and *T*_c_ versus pressure. The red, pink and blue solid circles represent *T*_c_ extracted from different runs of electrical resistance measurements, and the green triangles represent the *T*_c_ determined from the a.c. susceptibility measurements. The acronyms LMR and SC stand for the large magnetoresistant state and superconducting state, respectively. The error bars represent the s.d. (**b**) Hall coefficient (*R*_H_) as a function of pressure measured at 10 K and 1 Tesla, displaying a sign change from the positive to the negative at the critical pressure of 10.5 GPa. Solid purple circles and pink squares represent the *R*_H_ obtained from different runs. The inset shows the second derivative of the Hall coefficient, the maximum of which corresponds to the sign change of Hall coefficient. The shaded area indicates the pressure range where the superconductivity emerges and the sign of *R*_H_ changes.

A characteristic temperature (T*_ZF_) as the turn-on temperature of the LMR effect at zero field is defined, as indicated by the arrows in the inset of Fig. 3a. Such a definition of the T*_ZF_ is coincident with the temperature of the linear extrapolation of turn-on LMR temperatures under different magnetic fields. Then we summarize our experimental results in the pressure–temperature phase diagram (Fig. 6a). There are two distinct regions in the diagram: the LMR state and the superconducting state. It is found that the T*_ZF_ of the LMR state decreases with increasing pressure and vanishes at the critical pressure 10.5 GPa, where the superconductivity emerges at 2.8 K. The value of *T*_c_ increases up to a maximum at 13.0 GPa and then declines with further increasing pressure. This phase diagram clearly demonstrates how the pressure can effectively suppress the LMR state and induce superconductivity.

### Change of Hall coefficient with pressure

It is known that the perfect balance between hole and electron populations accounts for the ambient-pressure LMR effect^4^,^12^. To understand the suppression of the LMR effect under pressure, we conduct the *in situ* high-pressure Hall coefficient (*R*_H_) measurements at low temperature (Fig. 6b). The set-up of our high-pressure Hall measurements leads to the detected *R*_H_ combining signals from the two components of parallel and perpendicular to the tungsten chains (see Methods). Thus, the obtained results involve contributions from both hole and electron carries at the Fermi surface in the Brillouin zone. Below 10.5 GPa, the *R*_H_ is positive and it decreases with elevating pressure. On further increasing pressure, significantly, we find that the *R*_H_ suffers from a sign change from the positive to the negative at the critical pressure of 10.5 GPa, where the LMR state vanishes and superconductivity emerges.

## Discussion

The sign change in *R*_H_ is an indication of a significant reconstruction of Fermi surface, in good agreement with the reported Shubnikov-de-Hass oscillation measurements^13^. At the critical pressure, the second derivative of the Hall coefficient with respect to pressure shows a maximum, which is expected to become divergent in the zero temperature limit. Actually, our high-pressure XRD measurements indicate that, at the critical pressure, the *c* axis is compressed by 6.5%, almost 10-fold of the *a*-axis compressibility (0.6%) and twofold of the *b*-axis compressibility (3.3%). These results suggest that the reconstruction of Fermi surface is associated with anisotropic reductions of the lattice.

We thus propose that a quantum phase transition occurs at the critical pressure, separating the LMR state and superconducting state. Such a kind of quantum phase transition with the changes of the Fermi surface structure can be characterized by the Lifshitz phase transition^24^, so the emergence of superconductivity observed in the pressurized WTe_2_ may be connected with this transition, reminiscent of what is seen in Fe-based superconductors^25^. The mechanism of superconductivity in WTe_2_ deserves further investigations from both experimental and theoretical sides.

## Methods

### Single-crystal growth

Single crystals of WTe_2_ were grown by means of a solid-state reaction. Tungsten powder (99.9%) was mixed with excessive amounts of tellurium (99.999%) and placed in an alumina ampoule, then sealed in an evacuated quartz tube. The operation above was performed in a glove box filled with high-purity argon gas. The mixture was heated in a furnace to 1,000 °C with a rate of 100 °C h^−1^, followed by keeping the temperature at 1,000 °C for 5 h. After reaction, the sample was cooled down to 800 °C at a rate of 1 °C h^−1^, then to 700 °C at a rate of 5 °C h^−1^. The quartz tube was then taken out from the furnace, and put in a centrifuge to remove the Te flux.

### Experimental details of high-pressure measurements

Pressure was generated by a device, the so-called diamond anvil cell that consists of two opposing anvils sitting on the supporting plates. Diamond anvils of 300-μm flats were used for this study. Nonmagnetic rhenium gaskets with a 100-μm diameter hole were used for different runs of the high-pressure studies. The four-probe method was applied in the *ab* plane of the single-crystal WTe_2_ for all high-pressure transport measurements. For the high-pressure Hall coefficient measurements, the van der Pauw method was applied in this study. A constant current goes through the squared sample diagonally, and the Hall voltage is measured from the other diagonal. To keep the sample in a quasi-hydrostatic pressure environment, NaCl powder was employed as the pressure medium. The high-pressure alternating-current susceptibilities were detected using a primary/secondary-compensated coil system surrounding the sample^26^. High-pressure XRD experiments were performed at beam line 15U at the Shanghai Synchrotron Radiation Facility. A monochromatic X-ray beam with a wavelength of 0.6199 Å was chosen for all XRD measurements. Diamonds with low birefringence were selected for the experiments. To maintain the sample in a hydrostatic pressure environment, silicon oil was used as a pressure medium in the high-pressure XRD measurements. Pressure was determined by the ruby fluorescence method^27^.

## Additional information

**How to cite this article:** Kang, D. *et al*. Superconductivity emerging from a suppressed large magnetoresistant state in tungsten ditelluride. *Nat. Commun.* 6:7804 doi: 10.1038/ncomms8804 (2015).
